# Sphingosine 1-Phosphate Stimulates ER to Golgi Ceramide Traffic to Promote Survival in T98G Glioma Cells

**DOI:** 10.3390/ijms25158270

**Published:** 2024-07-29

**Authors:** Paola Giussani, Loredana Brioschi, Enida Gjoni, Elena Riccitelli, Paola Viani

**Affiliations:** Department of Medical Biotechnology and Translational Medicine, Università degli Studi di Milano, LITA Segrate, Via Fratelli Cervi, 93, 20054 Segrate, Italy; paola.giussani@unimi.it (P.G.); loredana.brioschi@unimi.it (L.B.); egjoni@ucsd.edu (E.G.); elenariccitelli@gmail.com (E.R.)

**Keywords:** ceramide, sphingosine-1-phosphate, ceramide transport, etoposide, glioblastoma cells

## Abstract

Glioblastoma multiforme is the most common and fatal brain tumor among human cancers. Ceramide (Cer) and Sphingosine 1-phosphate (S1P) have emerged as bioeffector molecules that control several biological processes involved in both cancer development and resistance. Cer acts as a tumor suppressor, inhibiting cancer progression, promoting apoptosis, enhancing immunotherapy and sensitizing cells to chemotherapy. In contrast, S1P functions as an onco-promoter molecule, increasing proliferation, survival, invasiveness, and resistance to drug-induced apoptosis. The pro-survival PI3K/Akt pathway is a recognized downstream target of S1P, and we have previously demonstrated that in glioma cells it also improves Cer transport and metabolism towards complex sphingolipids in glioma cells. Here, we first examined the possibility that, in T98G glioma cells, S1P may regulate Cer metabolism through PI3K/Akt signaling. Our research showed that exogenous S1P increases the rate of vesicular trafficking of Cer from the endoplasmic reticulum (ER) to the Golgi apparatus through S1P receptor-mediated activation of the PI3K/Akt pathway. Interestingly, the effect of S1P results in cell protection against toxicity arising from Cer accumulation in the ER, highlighting the role of S1P as a survival factor to escape from the Cer-generating cell death response.

## 1. Introduction

Glioblastoma multiforme (GBM) is the most common malignant primary brain tumor in adults and one of the most lethal human cancers. Furthermore, GBM is characterized by elevated resistance to radiation and cytotoxic chemotherapy [1,2], with median survival ranging from 12–15 months [3,4,5] despite aggressive surgical resection and conventional therapy.

Sphingolipids have been recognized as bioeffector molecules closely related to tumors, playing a critical role in the initial and developmental stages of cancer as well as in response to anticancer therapeutics [6,7,8,9,10]. Ceramide (Cer) is the central molecule of sphingolipid metabolism and several enzymes of Cer metabolism have been shown to be involved in regulating its levels [6,7]. It is synthesized in the endoplasmic reticulum (ER) and transported to the Golgi apparatus, where it is subsequently converted to sphingomyelin (SM), glucosylceramide (GlcCer) and more complex glycosphingolipids (GSLs) [6,7,11,12]. It is known that Cer is transported from the ER to the Golgi apparatus through two pathways: a protein-mediated transport specific for SM formation, carried out by CERT (the soluble ceramide transfer protein) [13,14,15,16,17,18], and a vesicular traffic (CERT-independent) for the biosynthesis of SM and/or GlcCer [14,15,16,19]. The two Cer transport mechanisms coexist separately, contributing to the regulation of Cer metabolism and levels in cells.

Cer acts as a tumor suppressor lipid through the inhibition of cell growth, induction of cell death, and/or modulation of senescence; moreover, Cer can act as a major player in the mechanism of action of many chemotherapeutic drugs [8,20,21,22,23,24]. Interestingly, Cer levels in human gliomas are inversely correlated with their malignant progression and poor prognosis, indicating that the malignant features of these tumors may be caused by alterations in ceramide metabolism [25,26,27,28].

On the other hand, sphingosine 1-phosphate (S1P), an intermediate of sphingolipid catabolism [29], has an important opposite role to that of ceramide, promoting activities strictly related to cancer progression, such as cell growth, invasiveness, cell survival, and angiogenesis. To regulate these processes, S1P acts by binding to five different G-protein-coupled receptors (S1PR1–5). S1P exerts its pleiotropic cellular actions because of the distinct expression patterns of S1PRs that, in turn, have downstream specific multiple intracellular signaling pathways [6]. Several studies have demonstrated that alterations in S1P levels of S1PRs and S1P metabolizing enzymes are involved in GBM pathophysiology [30]. In fact, it has been documented that S1P enhances proliferation, motility, invasiveness, and malignant behavior of glioblastoma cells [31,32,33,34,35].

Furthermore, the inhibition of Cer accumulation in the cell, as well as the activation of S1P signaling, have been implicated in the development of resistance to drug-induced apoptosis and escape from cell death [36,37,38,39,40].

Several lines of evidence indicate that, in glioma cells, accumulation of Cer in the ER is functional to the cytotoxic effects of different anticancer treatments [28,41]. Thus, the transport of Cer from ER to the Golgi apparatus in the biosynthesis of complex sphingolipids can represent a key pathway for limiting Cer accumulation in the ER. We previously demonstrated that this pathway is positively regulated by the pro-survival pathway phosphatidylinositol 3-phosphate kinase (PI3K)/Akt [42], a known mediator of extracellular S1P effects.

Based on these premises, the aim of this study was to investigate in T98G glioma cells, which are wild type for phosphatase and tensin homolog (PTEN), the physiological switch-off signal of PI3K/AKT, the effect of extracellular S1P on Cer metabolism and traffic, and the effect of S1P on the cytotoxicity induced by treatments that promote Cer accumulation in the ER.

## 2. Results

### 2.1. Role of Sphingosine-1-Phosphate in the Regulation of the PI3K/Akt Pathway

**Figure 1:**
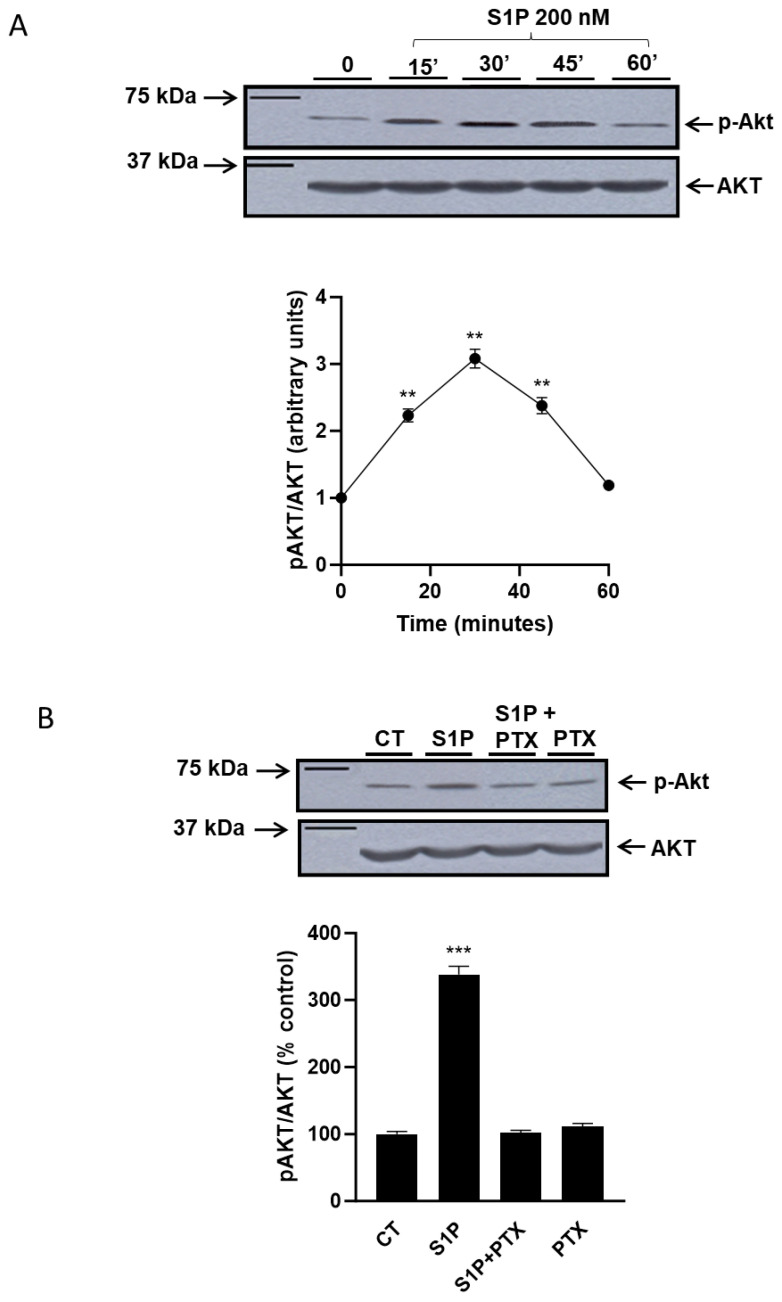
Effect of S1P and PTX on AKT phosphorylation in T98G glioma cells. T98G cells were serum-starved for 24 h and then: (**panel A**) incubated for the indicated times in serum-free medium at 37 °C in the presence or absence (control) of 200 nM S1P; ** *p* < 0.01 versus time 0 (one-way ANOVA followed by Tukey’s post hoc test); (**panel B**) pre-treated in serum-free medium for 2 h with 100 ng/mL (100 μM) PTX. Then, 200 nM S1P was added for 30 min. Cells were harvested for immunoblot analysis of phospho-AKT and AKT levels. All values are the mean ± S.D. of at least three individual experiments. *** *p* < 0.001 versus CT (one-way ANOVA followed by Tukey’s post hoc test).

First, we evaluated if S1P was able to induce the activation of the PI3K/Akt pathway in the T98G glioma cell line (Figure 1A). In T98G, the PI3K/Akt pathway is not constitutively activated and treatment with 200 nM S1P is able to stimulate the phosphorylation of Akt in time-dependent manner (Figure 1A): in particular, already after 15 min of treatment with S1P, phosphorylated Akt (pAkt) levels are increased, and the maximum activation of Akt was observed 30 min after S1P administration, with an increase of approximately three times compared to control. At longer times of treatment, we observed a progressive reduction in the levels of p-Akt to return to the baseline levels after 1 h (Figure 1A). Different concentrations of S1P (from 50 nM to 300 nM) promote an increase in pAKT similar to that shown in Figure 1 after 30′ treatment with 200 nM S1P.

In addition, data obtained stimulating cells with S1P in the presence of the pertussis toxin (PTX), a Go/Gi inhibitor (Figure 1B), support that also, in T98G cells, S1P activates the PI3K/Akt pathway through Go/Gi protein-coupled receptors [26,27].

### 2.2. Role of S1P on Cer Utilization for Complex Sphingolipids Synthesis 

In order to investigate the effect of S1P on Cer metabolic use in the biosynthesis of complex sphingolipids, we carried out a pulse and chase study, using tritiated sphingosine ([C3-^3^H]Sph) as a precursor of sphingolipids since [^3^H]Sph is efficiently internalized and rapidly incorporated first into Cer, and then, in SM and glycosphingolipids (GSLs) [43].

T98G cells were pulsed with [^3^H]Sph for 1 h and then chased in the presence of 200 nM S1P; at different times of chase (15′-30′-45′-60′) cells were harvested, and sphingolipid was extracted and analyzed.

**Figure 2:**
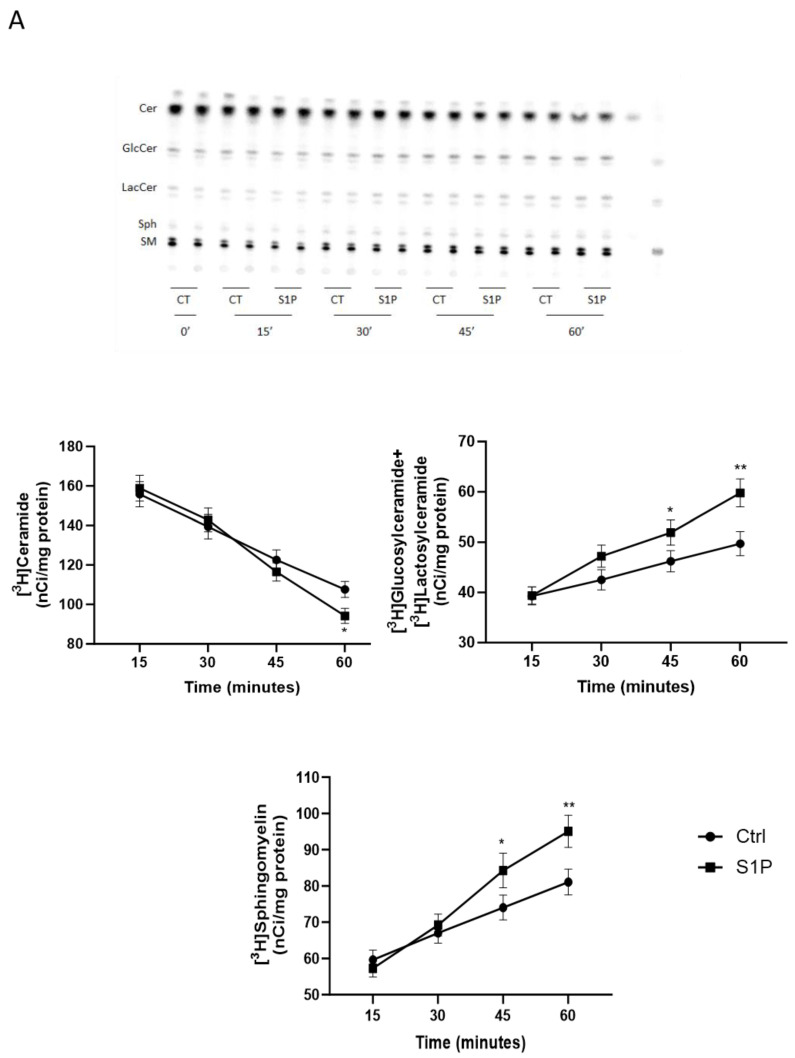

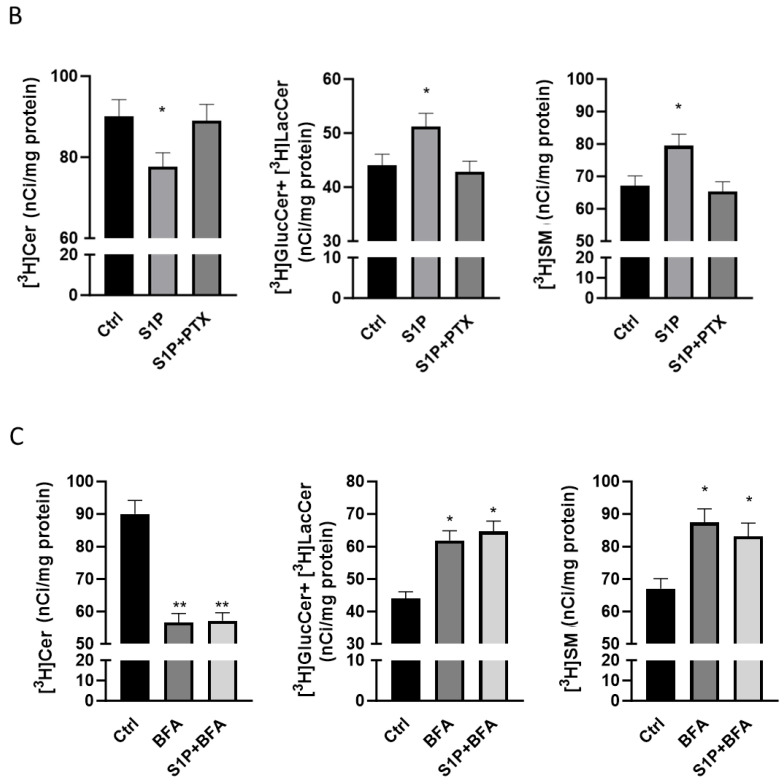
Effect of S1P on [^3^H]Sph inT98G glioma cells treated with or without PTX and BFA. T98G cells were serum-starved for 24 h and then: (**panel A**) pulsed with 0.15 μCi/mL [C3-^3^H]Sph for 1 h; (**panel B**) incubated in serum-free medium in the absence or presence of 100 ng/mL PTX. After 2 h, cells were pulsed with 0.15 μCi/mL [C3-^3^H]Sph for 30 min (**panel C**) and then pulsed with 0.15 μCi/mL [C3-^3^H]Sph for 30 min at 37 °C in the absence or presence of 1 μg/mL BFA. Then the cells were chased for (**panel A**) the indicated times (**panel B**, **C**) for 1 h, at 37 °C in the presence or absence of 200 nM S1P in the absence or presence of (**panel B**) 100 ng/mL PTX; (**panel C**) 1 μg/mL BFA. Radioactivity associated with each lipid was determined. (**panel A**) HPTLC plate. Values are expressed as nCi/mg protein (**panel A**–**C**). All values are the mean ± S.D. of at least three individual experiments performed in triplicate. * *p* < 0.05 versus Ctrl; ** *p* < 0.01 versus Ctrl (one-way ANOVA followed by Tukey’s post hoc test).

The results obtained (Figure 2A) show that in T98G cells treated with 200 nM S1P, there was an increase in the radioactivity associated with [^3^H]SM and [^3^H]glycosphingolipids and a concomitant decrease in the radioactivity associated with [^3^H]Cer.

In particular, the greatest increase in radioactivity associated with SM and glucosylceramide+lactosylceramide is observed after 60 min of chase in the presence of S1P. The treatment induced an 18% and 19% increase, respectively, of [^3^H]SM and [^3^H]glucosylceramide ([^3^H]GlucCer)+[^3^H]lactosylceramide ([^3^H]LacCer) (representative of [^3^H]glycosphingolipids in the organic phase) compared to the control. In parallel, at the same time of the chase, S1P leads to a 14% decrease in the levels of radioactivity associated with [^3^H]Cer. Treatment with 200 nM S1P does not cause significant changes in the levels of radioactivity associated with [^3^H]Sph.

Overall, the results suggest a role of S1P in the modulation of Cer metabolic use directed to the synthesis of both SM and GlucCer.

To test whether the effect exerted by S1P was receptor-mediated, we evaluated the effect of S1P on Cer metabolism in the presence of the G_i_/G_o_ protein-coupled receptor inhibitor pertussis toxin (PTX). The results obtained (Figure 2B) show that in the presence of PTX, S1P is no longer able to change the distribution of radioactivity associated with various sphingolipids.

Overall, these results indicate that the effect of S1P on modulating the Cer metabolic use directed to the biosynthesis of complex sphingolipids is due to its activity as an extracellular signaling molecule through its Go/i coupled receptors.

### 2.3. Role of S1P on Cer Transport in the ER-Golgi District

To obtain evidence on the role of S1P in the modulation of Cer trafficking from the ER to the Golgi apparatus for the complex sphingolipid synthesis, we investigated the effect of S1P on Cer metabolism in T98G cells treated with Brefeldin A (BFA).

BFA is a fungal metabolite that causes persistent inactivation of Arf GTP-ase-1, which is crucial in mediating the formation of an intermediary compartment ER-Golgi (ERGIC) that gives rise to the Golgi apparatus. It then promotes a collapse of the Golgi apparatus that fuses its membrane with that of ER, determining a condition in which Cer does not need any transport system to be available as a substrate for the Golgi localized GlcCer-synthase and SM-synthase.

Administration of BFA in pulse-chase experiments with [^3^H]Sph results in a 35% decrease in the radioactivity associated with [^3^H]Cer and an increase of 30% and 49%, respectively, in the radioactivity associated with [^3^H]SM and [^3^H]glucosylceramide+[^3^H]lactosylceramide compared to control, thus indicating a more efficient synthesis of complex sphingolipids.

In BFA-treated cells, S1P is no longer able to induce any significant change in the levels of radioactivity associated with the different tritiated sphingolipids compared to BFA-treated cells. These data suggest that the S1P effect on Cer metabolism is exerted through the regulation of Cer transport from the ER to the Golgi apparatus (Figure 2C). 

**Figure 3:**
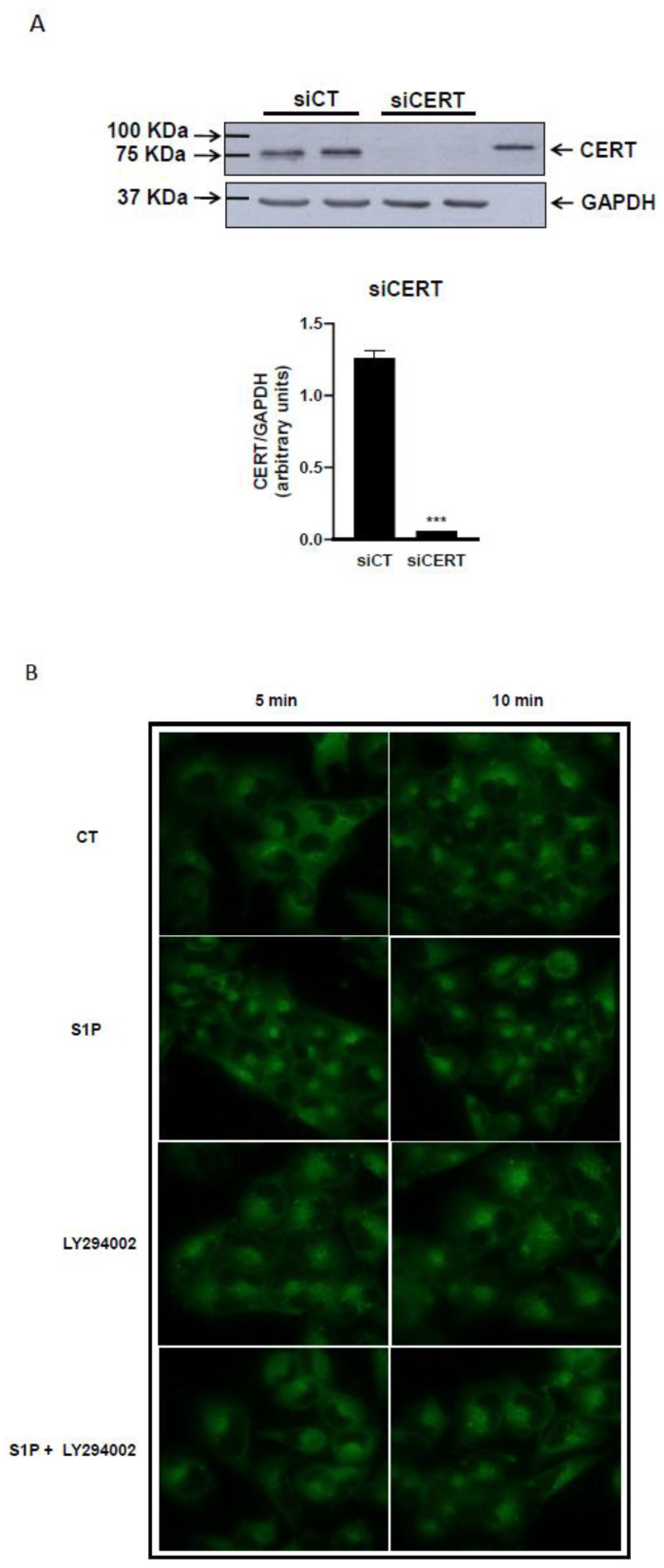
Effect of CERT downregulation on the intracellular distribution of BODIPY-C_5_Cer in T98G glioma cells. Cells grown in DMEM supplemented with 10% FCS were transfected with a mix of H87 and H424 siRNA for CERT (siCERT) and the corresponding non-targeting NT87 and H424 as control (siNT) as described in Mat. and Meth. (**Panel A**) the cells were analyzed for CERT levels. 72 h after transfection, cells were washed twice with PBS and harvested. Cell lysates (20 μg of protein) from two different preparations of siNT and siCERT transfected cells were analyzed by immunoblotting with a polyclonal anti-CERT antibody and polyclonal anti-GAPDH antibody. *** *p* < 0.001 versus siCT cells (one-way ANOVA followed by Tukey’s post hoc test). (**Panel B**) the cells were analyzed for fluorescence distribution. T98G glioma cells grown on a coverslip were transfected for silencing as previously described and 48 h after transfection, cells were pre-treated in serum-free medium at 37 °C for 30 min with or without 200 nM S1P and/or 10 μM LY294002 and then, cells were incubated with 2.5 μM BODIPY-C_5_Cer for 30 min at 4 °C. Labeled cells were further incubated at 37 °C for the indicated times with or without 200 nM S1P in the presence or absence of 10 μM LY294002 in order to allow the intracellular redistribution of fluorescent ceramides. Images are representative of at least five different experiments and were identically processed and printed.

Moreover, we evaluated the effect of S1P specifically on Cer vesicular traffic. To this purpose, we performed a microscopy fluorescence study using the Cer analog BODIPY-C_5_Cer in CERT-silenced cells. In these experiments, the level of silencing of CERT in T98G cells, evaluated by immunoblotting was ˃90% (Figure 3A). We evaluated the kinetics of transport of the BODIPY-C_5_-Cer, which mimics the behavior of natural Cer [44], between the ER and the Golgi apparatus after treatment with 200 nM S1P, in T98G cells silenced for CERT (Figure 3B).

This fluorescent Cer is internalized at the plasma membrane and then mainly distributed in the ER. From this district, using the transport systems of natural Cer, it is transported to the Golgi apparatus and used for the synthesis of complex sphingolipids. The images acquired at time 0 min show that the fluorescence is localized at the plasma membrane. Images acquired after 5 min of incubation at 37 °C show that in cells treated with S1P, fluorescence accumulated mainly at the Golgi apparatus (Figure 3B). In fact, when T98G cells were treated for 5 min without or with S1P, fluorescence accumulated mainly at the Golgi apparatus. The results show that S1P modulates the vesicular transport of Cer by increasing the speed of Cer traffic from the ER to the Golgi apparatus.

Next, we examined whether the effect of modulation of Cer vesicular transport by S1P was mediated by the PI3K/Akt signal transduction pathway. To this purpose, we analyzed the distribution of BODIPY-C_5_-Cer in T98G cells pre-treated with the PI3K/Akt inhibitor LY294002 in the presence or absence of S1P. In both cases, the accumulation of fluorescence at the Golgi is strongly reduced at all experimental times considered compared to control cells and S1P-treated cells (Figure 3B). In cells treated with LY294002 in the presence or absence of S1P, fluorescence accumulated at the Golgi apparatus (Figure 3B upper panel). Altogether, the results obtained demonstrate that S1P, through PI3K/Akt activation, increases the rate of Cer vesicular transport from the ER to the Golgi apparatus, promoting Cer removal from the ER direct to the synthesis of complex sphingolipids.

### 2.4. Role of Cer, Sphingosine-1-Phosphate and PI3K/Akt Pathway in the Etoposide-Induced Toxicity in T98G Cells

**Figure 4:**
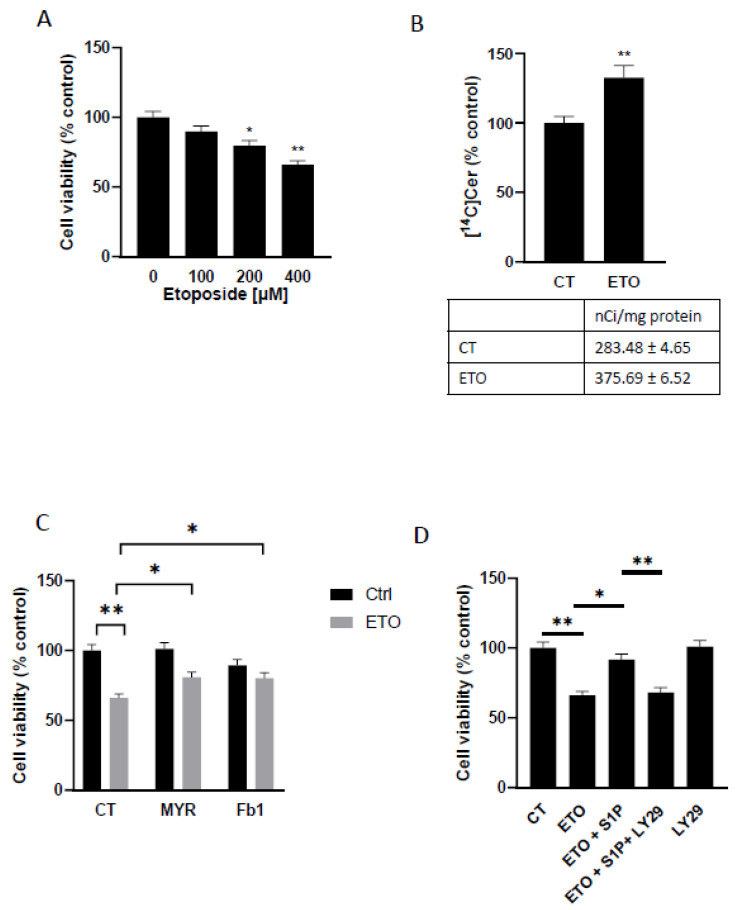
Effect of etoposide on ceramide biosynthesis and viability in T98G glioma cells and rescue activity of myriocin, fumonisin B1, and S1P on etoposide-induced cell toxicity. T98G cells were serum-starved for 24 h and then incubated in serum-free medium in the absence or presence of increasing doses of etoposide, and cell viability was assessed after 72 h of incubation (**panel A**), * *p* < 0.05 versus “0” (one-way ANOVA followed by Tukey’s post hoc test); ** *p* < 0.01 versus “0” (one-way ANOVA followed by Tukey’s post hoc test); serum-starved cells were pre-treated or not for 1 h with 400 µM etoposide (ETO), then pulsed with 3 μCi/mL [^14^C]Palmitate for 6 h with or without 400 μM etoposide, after that, radioactivity associated with each lipid was determined. Values are expressed as nCi/mg protein. All values are the mean ± S.D. of at least three individual experiments performed in triplicate (**panel B**), ** *p* < 0.01 versus CT (one-way ANOVA followed by Tukey’s post hoc test); serum-starved cells were incubated with 10 μM myriocin (MYR) or fumonisin B1 (Fb1) in the presence or absence of 400 μM ETO and cell viability was assessed 24 h later (**panel C**); starved cells were treated for 24 h with 400 μM etoposide alone, or together with 200 nM S1P, 200 nM S1P and 10 μM LY294002 (LY29) or 10 μM LY29 alone. (**panel D**) Cell viability was evaluated by the MTT assay. The viability of untreated cells was regarded as 100%. Mean ± S.D. is shown. (**Panel C**,**D**): One-way ANOVA with Tukey post hoc test. Data are presented as mean ± standard deviation ** *p* < 0.01; * *p* < 0.05.

Several lines of evidence indicate that also in glioma cells accumulation of Cer in the ER is functional to the cytotoxic effects of different anticancer treatments [8,20,21]. Among these drugs, etoposide has been reported to induce cytotoxicity; therefore, we evaluated its effect on T98G cell survival by comparing the survival rate after treatment with or without etoposide. To this purpose, we treated the cells with increasing doses of etoposide (ranging from 100 to 400 µM) for 72 h, and we performed the MTT assay to evaluate cell viability. As shown in Figure 4A, the treatment with 100 µM etoposide for 72 h did not significantly impair cell viability in T98G cells, while treatment with a higher dose, up to 400 µM, significantly reduced cell survival in a concentration-dependent manner; 20%, and 34% decrease in cell viability was observed at 200, and 400 µM, etoposide, respectively (Figure 4A).

Etoposide has been reported to induce Cer accumulation in the ER due to the activation of Cer biosynthesis [45]. Therefore, we evaluated if 400 µM etoposide was able to induce Cer accumulation in our cellular model. We performed a pulse metabolic study, using radioactive Palmitate ([^14^C]Palm) as a precursor of de novo synthesis of sphingolipids; in fact, [^14^C]Palm is efficiently internalized and rapidly metabolized to form Cer. T98G cells pre-treated or not for 1 h with 400 µM etoposide were then incubated with [^14^C]Palm for 6 h with or without etoposide. The results obtained (Figure 4B) show that in T98G cells treated with 400 µM etoposide, there was a 32.5% increase in the radioactivity associated with [^14^C]Cer, validating the data in the literature demonstrating Cer accumulation due to increased Cer biosynthesis.

To study if Cer accumulation at the ER was implicated in the toxic effect of etoposide, we evaluated the cell viability of T98G cells after treatment with etoposide in the presence or absence of Cer biosynthesis inhibitors. We used two different enzyme inhibitors: myriocin (MYR), a direct-to-serine palmitoyltransferase enzyme involved in the de novo pathway, or Fumonisin B1 (Fb1), direct to Cer synthase enzyme, involved in both salvage and in de novo pathway. The results in Figure 4C show that 24 h treatment with 400 µM etoposide determines a decrease in cell viability of 34% with respect to control cells. Treatment with a sub-toxic dose of MYR, together with 400 µM etoposide, caused a 14% decrease in cell survival, indicating that MYR is able to significantly reduce the toxic effect of etoposide. Furthermore, cells treatment with a sub-toxic dose of Fb1 together with 400 µM etoposide (Figure 4C) demonstrates that Fb1 is able to almost revert the toxic effect of etoposide when compared to cells treated with Fb1 alone, which induces a cell viability reduction of 16%.

These results suggest that Cer accumulation in the ER is a relevant mechanism involved in the toxicity of this molecule in T98G glioma cells.

We therefore evaluated if the activation of Cer traffic, promoted by S1P-dependent PI3K/Akt activation, could affect the viability of etoposide-treated cells. To this end, cell survival was assessed after treatment with etoposide in the presence or absence of S1P and/or LY294002, a specific inhibitor of PI3K. The results show that after 24 h, 200 nM S1P significantly reduced Etoposide toxicity, and LY294002 almost completely abolished the protective effect of S1P on cell death induced by etoposide; treatment with LY294002 alone does not cause significant changes in cell survival (Figure 4D).

These results demonstrate that S1P, activating the PI3K/Akt signal, promotes Cer transport and metabolism, protecting cells from the toxicity induced by Cer accumulation at the ER.

## 3. Discussion

GBM is a highly aggressive brain tumor with a poor prognosis and treatment prospects largely unchanged over the last 30 years. Among the signaling pathways altered in GBM, also Cer and S1P, which are known to play an opposite role in the regulation of glioma cell fate promoting cell death and cell survival, respectively, are established to play critical roles in GBM pathophysiology and therapeutics, and therefore, they are currently being studied as promising targets able to increase the effect of the standard treatment [46].

Cer and S1P are metabolically interconnected as they can be generated from the same precursor sphingosine: Cer in the biosynthetic salvage pathway, estimated to contribute from 50% to 90% of sphingolipid biosynthesis [47,48], and S1P in the catabolic process of sphingolipids. Thus, depending on the relative position of the Cer/S1P rheostat, the cells can be directed towards either cell death or survival by agents able to regulate the metabolic interconversion of ceramide-sphingosine-S1P. In addition, an intriguing aspect is the possibility that Cer and S1P-activated signaling pathways could also reciprocally regulate the levels and activity of these sphingoid mediators. 

In the present study, we demonstrated that in T98G glioblastoma cells, S1P binding to its receptors present at the plasma membrane is able to activate the PI3K/Akt signaling pathway. These results are in agreement with the literature that indicates S1P as a potent onco-promoter molecule in glioblastomas [31,32,49] with an autocrine/paracrine effect. Glioblastoma cells do express S1P receptors, mainly S1P_1_, S1P_2_, and S1P_3_, ref. [50] that promote cell proliferation, migration, and invasiveness [32,49] and S1P acts in different cell types, including glioma cells, also activating the PI3K/Akt pathway [51,52,53]. Furthermore, in our previous papers [42,54], we pointed out that the PI3K/Akt pathway is involved in the regulation of Cer vesicular transport from the ER to the Golgi apparatus. In this investigation, we demonstrated that, in T98G glioma cells, S1P actually increases the rate of Cer vesicular flow from the ER to the Golgi apparatus, favoring Cer metabolism toward complex sphingolipids biosynthesis through PI3K/Akt pathway stimulation. On the contrary, when S1P receptors are blocked by PTX action, S1P is no longer able to promote Cer utilization, indicating that the effect of S1P is due to its action as an extracellular mediator. Additionally, results obtained with BFA demonstrated that, in T98G glioma cells, the target of S1P action on Cer metabolism is the step of Cer transfer from ER to the Golgi apparatus. In fact, when cells were treated with BFA, which causes Golgi disassembly and redistribution to the ER, the conversion of Cer to both GlcCer and SM was strongly increased. The direct availability of Cer for SM and GlcCer synthases determined by BFA makes T98G glioma cells no more sensitive to S1P-dependent speeding of Cer utilization. This evidence further indicates that SM- and GlcCer-synthases are not the S1P target [42] and strengthens the notion that the S1P-speeding Cer utilization is mainly due to an effect on its translocation from ER to *cis*-Golgi. Moreover, in cells down-regulated for CERT, which represents a good model to study the vesicular transport of Cer, we obtained additional evidence that S1P can positively influence the intracellular vesicular traffic of Cer from ER to the Golgi apparatus, in agreement with previous data obtained in other cell types [55]. In fact, the analysis of BODIPY-C_5_-Cer intracellular distribution, which mimics the intracellular movements of natural Cer in T98G cells knocked down for the Cer transport protein CERT, shows that the fluorescence accumulation to the Golgi apparatus induced by S1P is reduced when the PI3K/Akt pathway is inhibited. This observation led us to hypothesize that S1P can increase the rate of vesicular transport of Cer from the ER to the Golgi apparatus, in agreement with data demonstrating that S1P positively regulates vesicular-mediated protein transport through the activation of PI3K/Akt.

Different lines of evidence indicate that both aberrant activation of the PI3K/Akt survival pathway [56,57] and downregulation of the death mediator Cer [25] play a critical role in the aggressive behavior, apoptosis resistance, and adverse clinical outcome of glioblastomas. Cer accumulation at the ER represents a mechanism through which different antineoplastic drugs, including etoposide, promote cell death. Cer accumulation has been proposed to induce ER stress in different cellular types [58,59]; in particular, in human glioma cells, it is demonstrated that the induction of de novo Cer synthesis by cannabinoid promotes ER stress, leading cells to apoptosis [41]. Different studies have suggested that drug resistance is often associated with the overcome of Cer-mediated apoptosis, and raising Cer levels may be helpful in treating cancer patients by getting over chemotherapeutic resistance [20,38,60,61].

Our research demonstrates that S1P protects cells from the toxic effect of etoposide that acts to stimulate Cer synthesis. In particular, there is evidence that in different cell types, as well as in glial cells, different chemotherapeutic drugs, including etoposide [8,20,21], induce cell death through the accumulation of Cer in the ER, a crucial subcellular site for the control of intracellular levels of this mediator. In fact, our data in T98G cells demonstrated that inhibition of Cer synthesis from both de novo and the recycling pathway significantly reduced the toxic effect of etoposide; furthermore, the PI3K inhibitor LY294002 completely abolished the protective effect of S1P on etoposide toxicity. These data demonstrate that the pro-survival action of S1P is mediated by the activation of the PI3K/Akt, which stimulates Cer transport from ER to the Golgi apparatus; through this mechanism, the toxic Cer accumulation in the ER was prevented, allowing escape from cell death.

The ability of the PI3K/Akt pathway to be regulated by/to regulate sphingolipid metabolism may be pathologically relevant in gliomas if we consider that the more malignant phenotypes of these tumors are associated with the up-regulation of the PI3K/Akt pathway [56,57], increased amounts of SM [62] of glucosylceramide [63], and low Cer levels [25]. Thus, the S1P-dependent Cer removal from the ER could participate in the molecular mechanisms involved in the regulation of Cer levels. Interestingly, Spiegel and Milstien, in their review in 2003 yet, discussed the role of S1P on Cer synthesis regulation [64]. They reported that the dephosphorylation of S1P is a relevant way to promote Cer synthesis from sphingosine in the salvage pathway. Our data demonstrate a new role of S1P in the regulation of Cer transport and metabolism. In agreement with Spiegel and Milstien, we could speculate that the pro-survival activity of S1P, controlling both Cer biosynthesis and traffic, might be the mechanism for its ability to suppress Cer-mediated cell death. Furthermore, S1P, by stimulating Cer metabolism, favors the maintenance of low Cer levels and promotes the biosynthesis of new membranes needed for cell proliferation and growth, key elements in tumor progression. Moreover, it is also tempting to speculate that Cer removal from the ER, as a consequence of an S1P-hyper-activated PI3K/Akt pathway, could also be involved in the refractoriness of glioma cells to Cer-based treatments, in particular those treatments that, in ER, act through Cer biosynthesis stimulation [8,65]. 

In conclusion, the results of the present study indicate that S1P, activating the PI3K/Akt signal, promotes Cer transport and metabolism, protecting T98G cells from the toxicity induced by Cer accumulation at the ER. The understanding of altered mechanisms in these tumors may help to shed light on possible targets for the development of more effective therapies.

## 4. Materials and Methods

### 4.1. Materials

All reagents were of analytical grade. Dulbecco’s modified Eagle’s medium (DMEM), Thiazolyl Blue Tetrazolium Bromide (MTT), fatty acid-free bovine serum albumin (BSA), Myriocin, Pertussis Toxin (PTX), Brefeldin A (BFA) and Etoposide were purchased from Sigma (St. Louis, MO, USA). Fetal calf serum (FCS) was from Cambrex (Walkersville, MD, USA). D-erythro-Sphingosine 1-Phosphate (S1P) and LY294002 were purchased from Enzo Life Sciences (Farmingdale, NY, USA). D-*erythro*-[3-^3^H]sphingosine (Sph) (21.2 Ci/mmol) [^14^C]Palmitate (Palm) was from PerkinElmer Life Science (Boston, MA, USA). High-performance thin layer chromatography (HPTLC) silica gel plates were from Merck (Darmstadt, Germany). N-(4,4,-difluoro-5-,7-dimethyl-bora-3a,4a-diaza-s-indacene-3-pentanoyl) sphingosine (BODIPY-C_5_Cer) was from Invitrogen-Molecular Probes (San Giuliano Milanese, Italy). LipofectAMINE 2000^®^ and the Stealth RNAi were from Invitrogen (Carlsbad, CA, USA). The primary antibodies recognizing Phospho-Akt (Ser473), AKT and CERT were from Cell Signaling Technology, Inc. (Danvers, MA, USA) and Bethyl Laboratories (Montgomery, TX, USA), respectively. The primary antibody recognizing GAPDH and the secondary anti-rabbit HRP-conjugated antibody was from Santa Cruz Biotechnology (Santa Cruz, CA, USA). SuperSignal WestPico Chemioluminescent Substrate and SuperSignal WestFemto Maximum Sensitivity Substrate were from Thermo Scientific (Rockford, IL, USA).

### 4.2. Cell Culture

We used the human glioma cell line T98G derived from glioblastoma, which was purchased from the American Tissue Culture Collection (Rockville, MD, USA) and cultured as recommended by the supplier. Cells were grown at 37 °C in DMEM supplemented with 10% FCS, 1 mM Na-pyruvate, 2 mM l-glutamine, 100 U/mL penicillin, 100 μg/mL streptomycin and 0.25 μg/mL amphotericin B in a fully-humidified incubator containing 5% CO_2_ and 95% air. The cells were starved of serum before the treatments and thereafter kept under serum-free conditions.

### 4.3. Treatment of Cultured T98G Cells

For cell treatments, stock solutions were prepared by dissolving the different molecules as follows: etoposide and LY294002 in dimethyl sulfoxide (DMSO); S1P in PBS with 4 mg/mL fatty acid-free bovine serum albumin (FAF-BSA); PTX and Fumonisin B1 (Fb1) in H_2_O; Brefeldin A (BFA) in ethanol, and Myriocin (MYR) in methanol. Stock solutions were diluted extemporaneously in fresh medium at the desired concentrations and administered to cells in the absence of Amphotericin B for the indicated periods of time. In each experiment, cells were also incubated with vehicles as controls and fatty acid-free BSA, ethanol, methanol, and DMSO had no effect on cell growth and viability.

### 4.4. Immunoblotting

*Akt-* In order to detect Akt phosphorylation, the cell extracts (40 μg proteins) were analyzed by means of Western blotting. In brief, the T98G glioma cells were lysed with Akt buffer (20 mM Tris-HCl pH 7.4, 150 mM NaCl, 1% NP-40, 10 mM sodium fluoride, 1 mM EDTA, 10 mM Na_4_P_2_O_7_, 1 mM Na_3_VO_4_, 2 μg/mL pepstatin, 2 μg/mL leupeptin, 2 μg/mL aprotinin). Solubilized proteins were centrifuged at 14,000× *g* at 4 °C for 10 min. Supernatants were subjected to 10% SDS polyacrylamide gel electrophoresis and transferred to nitrocellulose membranes. Membranes were blocked for 1 h at room temperature in Tris-buffered saline (10 mM Tris-HCl, pH 7.4, 140 mM NaCl) containing 0.1% Tween-20 (TBS-T) and 5% skimmed milk and then incubated with primary antibodies against phospho-Akt or Akt overnight at 4 °C. Membranes were washed in TBS-T, and bound antibodies visualized with horseradish peroxidase-coupled secondary antibodies (Immunopure) and chemiluminescent substrate. The relative intensities of bands were quantified by densitometry.

*CERT-* CERT immunoblotting was performed using si-control and si-CERT transfected cells lysed with CERT buffer (10 mM Tris-HCl pH 7.4, 0.25 M sucrose, 0.5 mM phenylmethylsulfonyl fluoride, 1 μg/mL aprotinin, 1 μg/mL leupeptin, 1 μg/mL pepstatin), processed and analyzed as previously described [14].

### 4.5. RNA Interference

Small interfering RNA (siRNA) duplexes for human CERT were obtained with the BLOCK-iT™ RNAi Designer from Invitrogen. The selected sequences were S87: 5′-CCUCAGUAAGUGGACAAACUACAUU-3′, S424: 5′-GGCUACUCUGCAACAUCCACCUCUU-3′. The siRNA control is a non-targeting scrambled sequence of S87 and S424 oligonucleotides (NTS87; NTS424). The lack of targeting for other T98G genes by the designed siRNA duplexes was then checked by BLAST to avoid silencing of multiple genes other than CERT. T98G glioma cells plated at 1.5 × 10^4^ cell/cm^2^ were maintained 24 h in DMEM plus 10% FCS and then transfected in the same medium with 100 nM (final concentration) of S87 + S424 mix (1:1 by mol) and the non-targeting corresponding sequences using Lipofectamine 2000 according to the manufacturer’s protocol. The experiments were performed 72 h or, when indicated, 48 h after transfection. Specific silencing achieved was evaluated by immunoblotting.

### 4.6. [^3^H]Sphingosine Metabolism

T98G cells plated at 1.5 × 10^4^ cell/cm^2^ were grown for 24 h in DMEM plus 10% FCS, serum-starved for 24 h, and then pulsed with 0.15 μCi/mL [C3-^3^H]Sph for 1 h and at the end chased for the indicated times at 37 °C in the presence or absence of 200 nM S1P. Otherwise, T98G cells were serum-starved for 24 h and then incubated in a serum-free medium in the absence or presence of 100 ng/mL PTX for 2 h. Then, cells were pulsed with 0.15 μCi/mL [C3-^3^H]Sph for 30 min in the presence or absence of 1 μg/mL BFA and chased for 1 h in the presence or absence of 200 nM S1P and/or 100 ng/mL PTX and/or 1 μg/mL BFA. In the end, cells were washed twice with PBS, harvested, and submitted to lipid extraction and partitioning as previously described [66]. The methanolized organic phase was analyzed by HPTLC using chloroform/methanol/water (110/40/6 by vol) as a solvent system. Digital autoradiography of HPTLC plates was performed with Beta-Imager 2000 (Biospace, Nesles-la-Vallée, France) and the radioactivity associated with individual lipids was measured using the software provided with the instrument. The [^3^H]labeled sphingolipids were recognized and identified as previously described [66]. 

### 4.7. [^14^C]Palmitate Metabolism

T98G cells plated at 2 10^4^ cell/cm^2^ were grown 24 h in DMEM plus 10% FCS, serum-starved for 24 h, treated 1 h with 400 µM etoposide, and then pulsed with 3 μCi/mL [^14^C]Palmitate for 6 h at 37 °C in the presence or absence of 400 µM etoposide. In the end, cells were washed twice with PBS, harvested, and submitted to lipid extraction and partitioning as previously described [66]. The methanolized organic phase was analyzed by HPTLC using chloroform/methanol/2 M NH_4_OH (40/10/1 by vol) as a solvent system. Digital autoradiography of HPTLC plates was performed with Beta-Imager 2000 (Biospace, France) and the radioactivity associated with individual lipids was measured using the software provided with the instrument.

### 4.8. Analysis of the Intracellular Distribution of Fluorescent Ceramides

T98G cells plated at 1.5 × 10^4^ cell/cm^2^ were grown on glass coverslips and transfected as described in the Section 4.5. 24 h after transfection, cells were placed in DMEM without FCS for 24 h. First, cells were pre-treated with 200 nM S1P in the presence or absence of 10 μM LY294002 for 30 min in DMEM without FCS at 37 °C; then, they were incubated with 2.5 μM BODIPY-C_5_-Cer (as 1:1 complex with fatty acid-free BSA) in DMEM at 4 °C for 30 min [14]. Then, cells were washed with DMEM without FCS in the presence of 4 mg/mL fatty acid-free BSA and treated with 200 nM S1P in the presence or not of 10 μM LY294002 for the indicated times at 37 °C. Cells were then washed (three times with PBS) and fixed with 0.5% glutaraldehyde solution in PBS for 10 min at 4 °C. The specimens were immediately observed and analyzed with a fluorescence microscope (Olympus BX-50) (Olympus, Segrate, Italy) equipped with a fast high-resolution CCD camera (Colorview 12) and image analytical software (analySIS from Soft Imaging System GmbH Pro 3.2, Münster, Germany). 

### 4.9. MTT Assays

Cells were plated at 18,000 cells/cm^2^ and maintained in DMEM plus 10% of FCS for 24 h. Then, cells were placed in DMEM without FCS for 24 h and incubated in serum-free medium in the absence or presence of 400 μM etoposide and/or 10 μM myriocin and/or fuminisin B1, and/or 200 nM S1P for 24 h at 37 °C. At the end of treatments, cells were washed with PBS and incubated with 0.8 mg/mL of Thiazolyl Blue Tetrazolium Bromide (MTT) in DMEM without FCS at 37 °C, 5%CO_2_. After 4 h, cells were lysed in 2-propanol/formic acid (95:5), and absorbance was measured at 570 nm.

### 4.10. Other Methods

The total protein amount was assayed with the Coomassie Blue-based Pierce reagent, using bovine serum albumin fraction V as standard. Radioactivity was measured by liquid scintillation counting. The statistical significance of differences was determined by Student’s *t*-test.

## Data Availability

All data are available from the corresponding author upon reasonable request.

## Abbreviations

Cer	Ceramide
ER	Endoplasmic reticulum
ETO	Etoposide
GBM	Glioblastoma multiforme
GlcCer	Glucosylceramide
GSL	Glycosphingolipids
PTX	Pertuxin Toxin
SM	Sphingomyelin
Sph	Sphingosine
S1P	Sphingosine-1-phosphate
PI3K	phosphatidylinositol 3-phosphate kinase
CERT	ceramide transfer protein
BFA	Brefeldin A
Fb1	Fumonisin B1
MYR	Myriocin
